# A short-form of team mindset scale: Using psychometric properties of the items

**DOI:** 10.3389/fpsyg.2022.1063541

**Published:** 2023-01-04

**Authors:** Mirim Kim, Soo Jeoung Han, JiYoon Kim

**Affiliations:** ^1^BK21 Four R&E Center for Psychology, Korea University, Seoul, Republic of Korea; ^2^Graduate School of Education, Yonsei University, Seoul, Republic of Korea; ^3^Department of Education, Korea University, Seoul, Republic of Korea

**Keywords:** team mindset, scale development, construct validity, factor analysis, item analysis

## Abstract

As the importance of a team function in educational and industrial settings has been emphasized, practical knowledge of team mindsets can be useful. Previous studies extended the mindset concept of individuals to the team context and developed a 48-item team mindset scale (TMS). However, the original TMS had an issue regarding the length of the survey when using it with many other scales. Therefore, the present study tested the psychometric properties of 48 items under a variety of perspectives to suggest a shorter version of TMS measuring the perception of the team mindset and having the desirable characteristics. We examined the construct validity of the TMS with exploratory factor analysis (EFA) with target rotation in Study 1 and tested item-level psychometric properties of the TMS in Study 2 based on classical test theory (CTT) and item response theory (IRT). Based on the result, we suggested the short-form of TMS, an 8-item TMS with adequate psychometric characteristics.

## 1. Introduction

Drawing upon Carol Dweck’s theoretical framework of growth and fixed mindsets, most studies have explored if one’s mindset can impact other possible outcomes, such as academic results or performance of individuals (Ravenscroft et al., 2012; Pennington and Heim, 2016). Dweck (2006) uses the terms growth and fixed mindset to refer to the implicit beliefs people possess about the malleability of intelligence (Heslin and VandeWalle, 2008; Ravenscroft et al., 2012; Özduran and Tanova, 2017). According to Dweck (2012), fixed mindset individuals believe that human attributes are simply fixed traits; they accept their abilities as unchanging. Fixed mindset students are outcome-focused and screen out negative feedback. In comparison, growth mindset individuals believe that anyone can become substantially more intelligent through their effort and education, or that all people can change and develop their behavior, personality, or character over time (Dweck, 2012). Growth mindset students are process-focused, seeking and responding to feedback (Dweck, 2006).

Scholars have found that individual growth mindsets can increase academic performance (Ravenscroft et al., 2012; De Castella and Byrne, 2015; Pennington and Heim, 2016), help out to seek difficult challenges (Lee et al., 2012), enhance motivation for academic goals (Aditomo, 2015; Gheith and Aljaberi, 2017), promote feedback activities (Lee et al., 2012; Forsythe and Johnson, 2017), and support better relationships and willpower (Dweck, 2012). Thereby, many mindset studies have found the way to foster the growth mindset. Most of the studies have focused on the individual mindset; however, as the importance of teams in both workplaces and education settings has been emphasized, it would be practical and applicable knowledge to discuss team mindsets. Nevertheless, only few studies have contemplated team mindsets. The mindset studies about teams and the related characteristics remained under-studied (Han and Stieha, 2020; Han et al., 2022) even if a few studies have suggested possible connections between individual mindsets and team mindsets (e.g., Heslin et al., 2005; Gutshall, 2013; Özduran and Tanova, 2017).

Several theories propose the possibility of team mindsets. Relational theory values the idea that collectives are to be differentiated from an aggregated of individuals where they are ongoing relationships with team members rather than in the individual mind (Drath et al., 2008). In addition, the leader-member exchange theory supports team mindsets as it values the two-way relationship between members in a team, as each team member’s mindsets or behaviors can influence mutual trust and willingness to share their knowledge (Han et al., 2022). Although we found no studies that directly discuss the concept of team mindset, several studies show indicators of individuals’ mindsets impacting mindsets on the team. For example, Gutshall (2013) illustrates the importance of team members’ trust in others. Other studies suggest the importance of collective or group mindsets by examining the effects of managers’ mindsets (Heslin and VandeWalle, 2008; Özduran and Tanova, 2017). Heslin et al. (2005) found that managers with a growth mindset tend to conduct active coaching activities for their team members, leading to their organizational citizenship behaviors. These studies suggest a relationship between individual and collective/team mindsets through findings that leaders can influence others’ thoughts and outcomes. More studies are needed to examine the possibility of team mindsets representing teams rather than individuals.

As a recent team mindsets study, Han et al. (2019) have explored common themes regarding team growth and fixed mindset based on a qualitative study by conducting interviews and literature review. Based on their findings, team mindset scale (TMS) has been developed to grasp the team mindset themes *via* a quantitative method (Han and Kim, 2020). The concept of a team mindset has also been explored to examine if individual mindsets increase a team growth mindset and shared leadership (Han et al., 2022), which explored the possibility of the TMS being different from individual mindsets. The TMS is a questionnaire having 48 items measuring the team growth mindset and team fixed mindset; however, there is a limitation to using the original length of the test. Statistically, reliability and validity tend to be higher when a scale length is longer with more items (Ebel, 1967; McDonald, 1999; Lord and Novick, 2008). In practice, however, the longer survey is likely to derive fatigue, tiresomeness, or less motivation followed by low response rates. Therefore, it is significantly related to the low quality of the measurement (Hart et al., 2005; Andreadis and Kartsounidou, 2020) because multiple scales are usually combined in a single survey to measure different aspects of individuals simultaneously.

Based on this concern, the current study aims to develop a short version of TMS. Psychometric properties of the original team mindsets questionnaire were examined in a variety of aspects to reach the desired length of the scale. The other mindset scales, such as individual-level mindsets (Dweck, 1999) and creative mindsets (Karwowski, 2014), have a relatively shorter number of items as 8 and 10, respectively. Therefore, we proposed a similar length of a short-form of TMS through Study 1 and Study 2.

## 2. Study 1

In Study 1, the measurement properties of the 48-item were examined *via* exploratory factor analysis (EFA) with target rotation. The construct validity was investigated regarding the six themes (Han et al., 2019) and team mindset constructs.

### 2.1. Materials and methods

#### 2.1.1. Participants and data collection

Participants were recruited from a public research university in Spring 2019. We specifically stated that the target participants would be those who experienced team projects within 6 months because the TMS measured their perception of team’s mindsets. A total of 1,297 students responded to the original TMS, and Study 1 used 908 responses after dropping the incomplete surveys [Female: 62.2%, Caucasian: 79.7%, Age: 27.6_*mean*_, 10.47_*std*_, (18, 70)_*range*_].

#### 2.1.2. Measures team growth and fixed mindset questionnaire

Each team growth and fixed mindset was a 5-point Likert scale (1 = *Strongly Disagree* to 5 = *Strongly Agree*) of 24-item and asked about six sub-themes of the team mindset: (a) perseverance; (b) openness to change; (c) taking risk; (d) accepting feedback; (e) learning from mistakes; (f) learning from team. Four items measured each theme except for two themes: task risk (three items) and learning from the team (five items).

#### 2.1.3. Analysis

We supposed a certain number of factors to be estimated based on Han et al.’s (2019) findings about the six main themes under the two team mindsets. However, the factor structures were still tentative because the recent team mindset studies still need to be validated in a variety of settings. Therefore, we tested a 6-factor structure through the EFA with target rotation (Browne, 1972a,b) to reflect the hypothesized factor structure and explore the possible item-factor relationships. The EFA with target rotation fits the model with a certain number of factors as CFA and allows the cross-loadings as the general EFA (Browne, 1972b; Zhang et al., 2019). We identified the items representing the six themes first; the items not measuring their target theme were considered inadequate and screened out from further analysis. Additional factor analysis was conducted to examine item-team mindset relationships. All analyses were based on the maximum likelihood (ML) estimator through *Mplus* 8.4 (Muthén and Muthén, 1998–2017).

### 2.2. Results

#### 2.2.1. Exploratory factor analysis with target rotation

A 6-factor model representing six themes was tested first; factor loadings between items and the relevant theme were expected to be bigger than the other factor loadings between items and irrelevant themes. For example, eight items were to measure Perseverance-theme (four-item for each team growth and fixed mindset); the factor model of the Perseverance-theme was expected to be constructed with the eight items rather than with the other 40 items. The model fit of the 6-factor model was appropriate (comparative fit index (CFI) = 0.94, root mean square error of approximation (RMSEA) = 0.04, standardized root mean square residual (SRMR) = 0.03) compared to criteria (CFI > 0.95, RMSEA < 0.06, or SRMR < 0.05; Hu and Bentler, 1999). Therefore, we examined item properties given the factor analysis model and concluded some of the items as inappropriate; when their factor loadings were not statistically significant, or the targeted factor loadings were close to 0 which were expected to be greater than zero, or the item had the equal signs of factor loadings on both team growth and fixed mindsets indicating the same side of the continuum. In addition to such criteria, we re-evaluated item contents; therefore, 22 out of 48 total items were ruled out from further factor analysis.

With the remaining items, the 2-factor model representing the team growth and fixed mindsets was tested. The model fit was not good enough, and we found the source of a misfit from three team fixed mindset items: TFM_Risk3, TFM_Change3, and TFM_Feedback2. Their factor loadings were against the other team fixed mindset items, but in line with team growth mindset items. These items were excluded from the revised factor model for the team mindsets, and modification indices were considered for a better model fit. The modification indices suggested some unique factor variance correlations. We found that the two sets of items had a similar word but indicated different team mindsets; therefore, their unique factor correlations were estimated: TGM_Change4 with TFM_Change4; TGM_Feedback3 with TFM_Feedback3. The modified 2-factor model had an adequate global fit (CFI = 0.94, RMSEA = 0.06, SRMR = 0.03).

To sum up, Study 1 investigated construct validity regarding team mindsets and tested the item-construct associations. Based on the result, the 23 items in Table 1 showed appropriate psychometric properties: their factor loadings were higher than or close to 0.3 and the corresponding item contents represented individual’s perception of team’s mindsets.

**TABLE 1:** Factor loadings and factor correlation from exploratory factor analysis (EFA).

Themes	Item	Team growth mindset	Team fixed mindset
**Factor loadings**			
Perseverance	TGM_Perseverance1	**0**.**39**	−0.19
Open to change	TGM_Change1	**0**.**76**	0.13
	TGM_Change2	**0**.**64**	0.01
	TGM_Change3	**0**.**65**	0.20
	TGM_Change4	**0**.**73**	−0.01
	TFM_Change2	−0.04	**0**.**54**
	TFM_Change4	−0.38	**0**.**31**
Taking risk	TGM_Risk1	**0**.**68**	−0.16
	TGM_Risk2	**0**.**74**	−0.04
	TFM_Risk1	−0.10	**0**.**49**
Accepting feedback	TGM_Feedback2	**0**.**53**	0.25
	TGM_Feedback3	**0**.**73**	0.11
	TGM_Feedback4	**0**.**74**	0.04
	TFM_Feedback3	−0.12	**0**.**29**
Learning from mistakes	TGM_Mistake3	**0**.**50**	0.22
Learning from team	TGM_Desire1	**0**.**65**	−0.20
	TGM_Desire2	**0**.**68**	−0.17
	TGM_Desire3	**0**.**68**	−0.09
	TGM_Desire4	**0**.**57**	−0.11
	TGM_Desire5	**0**.**59**	−0.24
	TFM_Desire3	0.08	**0**.**70**
	TFM_Desire4	0.09	**0**.**6**
	TFM_Desire5	−0.26	**0**.**41**
**Factor correlation**			
TGM	1		
TFM	−0.58	1	

TGM, team growth mindset; TFM, team fixed mindset; bold indicates a factor loading between an item and a target construct.

## 3. Study 2

While Study 1 evaluated the construct validity of the team mindset items by using dimensional properties, Study 2 investigated item-level properties. The item-level analyses were conducted with two theoretical approaches: classical test theory (CTT) and item response theory (IRT). Later, results from Study 1 and Study 2 were considered together to set a short-form TMS.

### 3.1. Materials and methods

#### 3.1.1. Participants, data collection, and measures

Study 2 used empirical data from the workplace although the team mindset measures were identical to Study 1. The recruited participants were 400 team members within a department of public works that resided in a traffic division in the continental USA. Unfortunately, the participation rates were extremely low due to the unprecedented pandemic; therefore, Study 2 used 91 responses from the employees without missing on the team mindset measures [Male: 62%, White: 47.8%, Age: 40.9_*mean*_, 12.85_*std*_, (21, 80)_*range*_].

#### 3.1.2. Analysis

A complex model estimating many parameters is less likely to converge with a small sample size. A factor analysis needed to concern multiple team mindsets; however, the second sample was relatively small to test all relationships among 48 items and the two team mindsets due to the convergence issue (MacCallum et al., 1999). Therefore, we assumed unidimensionality of each team mindset and conducted item analyses. Each set of items within the team growth mindset and the team fixed mindset was separately analyzed. Under the CTT framework, item-total correlations for the item discrimination level, category response rates for checking flooring and ceiling effects, and reliabilities were evaluated. For the IRT analysis, we estimated item parameters and item information given a generalized partial credit model (GPCM; Muraki, 1992) using PARSCALE (Muraki and Bock, 1993) with an expected a posterior (EAP) estimator.

### 3.2. Results

#### 3.2.1. Classical test theory approach

Table 2 presents item properties for team growth and fixed mindsets. First, a Pearson correlation coefficient between the item and the total score was evaluated as the item discrimination index under the CTT framework. All items showed moderate or higher discrimination power based on the criteria (i.e., ρ 0.40; Ebel, 1965), except for an item in the team fixed mindset (TFM_Desire5). Second, the flooring and ceiling effects were examined by checking response rates in the first category (*Strongly Disagree*) and the last category (*Strongly Agree*). If most people responded to the first or the last category, such items might have detected the extreme level of team mindset only. 19 team growth mindset items and six team fixed mindset items showed an over 20% response rate in the last category. Lastly, we examined Cronbach’s alpha (α) coefficient and considered it acceptable when α was greater than or equal to 0.70 (Cortina, 1993). Both team growth mindset (α = 0.97) and team fixed mindset items (α = 0.91) showed appropriate test reliability.

**TABLE 2:** Item analysis of team growth and fixed mindset in classical test theory (CTT).

Item: team growth mindset	Item-total correlation	Item: team fixed mindset	Item-total correlation
TGM_Perseverance1	0.66	TFM_Perseverance1	0.53
TGM_Perseverance2	0.88	**TFM_Perseverance2**	0.56
TGM_Perseverance3	0.79	TFM_Perseverance3	0.55
TGM_Perseverance4	0.77	TFM_Perseverance4	0.58
TGM_Change1	0.87	**TFM_Change1**	0.50
TGM_Change2	0.87	TFM_Change2	0.65
TGM_Change3	0.83	**TFM_Change3**	0.61
TGM_Change4	0.80	TFM_Change4	0.45
TGM_Risk1	0.89	**TFM_Risk1**	0.67
TGM_Risk2	0.91	**TFM_Risk2**	0.47
TGM_Risk3	0.88	TFM_Risk3	0.60
TGM_Feedback1	0.84	TFM_Feedback1	0.65
TGM_Feedback2	0.54	TFM_Feedback2	0.68
TGM_Feedback3	0.83	TFM_Feedback3	0.78
TGM_Feedback4	0.83	TFM_Feedback4	0.72
TGM_Mistake1	0.86	TFM_Mistake1	0.41
TGM_Mistake2	0.53	TFM_Mistake2	0.44
TGM_Mistake3	0.52	TFM_Mistake3	0.66
TGM_Mistake4	0.84	TFM_Mistake4	0.51
TGM_Desire1	0.82	TFM_Desire1	0.51
TGM_Desire2	0.82	**TFM_Desire2**	0.48
TGM_Desire3	0.79	TFM_Desire3	0.52
TGM_Desire4	0.78	TFM_Desire4	0.72
TGM_Desire5	0.84	**TFM_Desire5**	0.32

*N* = 91; bold indicates an item having more than 20% of responses in the first or the last category.

#### 3.2.2. Item response theory approach

The items having low item discrimination and information were not preferred for the final set of TMS to keep the high level of precision of the test (Baker, 1985). Table 3 presents parameter estimates by fitting GPCM to team mindset items: item slope (i.e., item discrimination; a) and the item information.

**TABLE 3:** Item response theory (IRT) item parameters for 24 items on team growth and fixed mindset.

Item: team growth mindset	Slope (*a*)	Item information: I(θ)	Item: team fixed mindset	Slope (*a*)	Item information: I(θ)
TGM_Perseverance1	**0.395**	**0.221**	**TFM_Perseverance1**	**0.361**	**0.373**
TGM_Perseverance2	1.349	1.058	TFM_Perseverance2	1.017	1.517
TGM_Perseverance3	**0.611**	**0.512**	TFM_Perseverance3	0.806	1.144
TGM_Perseverance4	**0.768**	**0.630**	TFM_Perseverance4	0.675	1.341
TGM_Change1	1.541	1.464	TFM_Change1	0.871	1.203
TGM_Change2	1.131	1.184	TFM_Change2	0.692	1.275
TGM_Change3	0.860	0.778	TFM_Change3	1.017	1.403
TGM_Change4	0.894	0.938	**TFM_Change4**	**0.414**	**0.370**
TGM_Risk1	0.879	0.868	TFM_Risk1	0.900	1.874
TGM_Risk2	1.425	1.876	TFM_Risk2	0.657	1.028
TGM_Risk3	1.688	2.010	TFM_Risk3	0.691	1.118
TGM_Feedback1	0.842	0.685	TFM_Feedback1	0.998	1.864
TGM_Feedback2	**0.402**	**0.279**	TFM_Feedback2	1.131	1.863
TGM_Feedback3	1.328	0.873	TFM_Feedback3	0.889	1.307
TGM_Feedback4	0.985	0.845	TFM_Feedback4	0.956	1.605
TGM_Mistake1	**1.902**	**1.102**	**TFM_Mistake1**	**0.540**	**0.619**
TGM_Mistake2	**0.520**	**0.470**	**TFM_Mistake2**	**0.322**	**0.337**
TGM_Mistake3	0.413	0.249	TFM_Mistake3	0.824	1.444
TGM_Mistake4	1.466	1.283	**TFM_Mistake4**	**0.460**	**0.582**
TGM_Desire1	1.629	2.052	TFM_Desire1	0.985	2.062
TGM_Desire2	1.128	1.254	TFM_Desire2	0.677	0.939
TGM_Desire3	1.327	1.228	**TFM_Desire3**	**0.440**	**0.527**
TGM_Desire4	1.403	1.651	TFM_Desire4	0.731	1.403
TGM_Desire5	0.875	0.963	**TFM_Desire5**	**0.336**	**0.508**

*N* = 91; bold indicates an item having a low slope parameter and low item information.

With the moderate cut-off for the item slope parameter (0.65 ≤ a < 1.34; Baker, 1985), we identified the items to be dropped from the short-form TMS. In general, team growth mindset items had high slope parameter values ($\bar{a}\,1.07$); therefore, we applied the upper limit of the moderate item slope (1.34) to them to select the small number of items for the short-form TMS. On the other hand, the average slope parameter of team fixed mindset items was moderate ($\bar{a}\,0.73$), that is, the lower limit (0.65) of the cut-off value was used. The 16 items of the team growth mindset and seven team fixed mindset items had the slope parameters below the designated cut-off value, and they were not preferred for the short-form TMS.

The item slope and the item information were tightly related: an item with a low item slope will give less information on the target construct. Therefore, we also considered the item information of each item and preferred the item having the higher item information. The third and the last column of Table 3 present the item information of each item at the middle level of trait θ (team mindset θ = 0). The six team growth mindset items (TGM_Perseverance1,3 4, TGM_Feedback2, TGM_Mistake1,2) and the seven team fixed mindset items (TFM_Perseveranc1, TFM_Change4, TFM_Mistake1,2,4, TFM_Desire3,5) had relatively small information at the middle level of team mindset.

#### 3.2.3. Item selection for short-form of TMS

**FIGURE 1:**
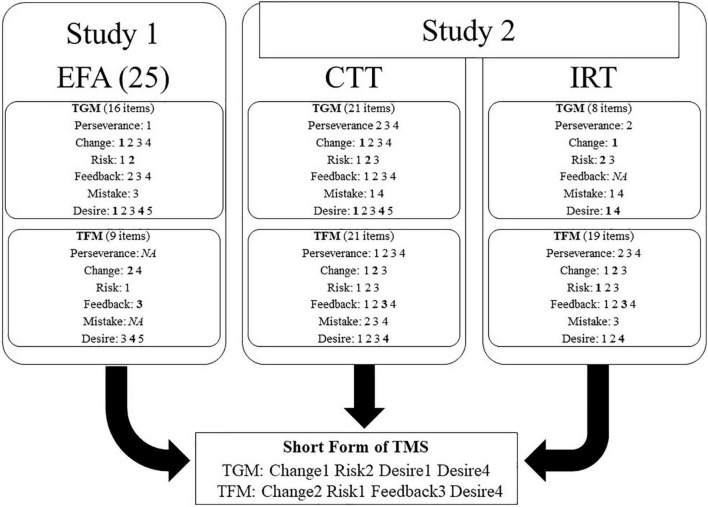
The process of item selection.

For the final set of items of short-form TMS, we took the two study results together and ruled out the items with less desirable psychometric properties across the two studies. Figure 1 represents the ruling-out strategy and the selected items. Study 1 tested the construct validity based on the dimensional aspects of the items, and Study 2 conducted the item-level analyses. The eight items were selected as they performed appropriately in common across the two studies. The selected items represented the team mindsets well based on Study 1 and had moderate or high discrimination power contributing to the high information on the scale based on Study 2.

**FIGURE 2:**
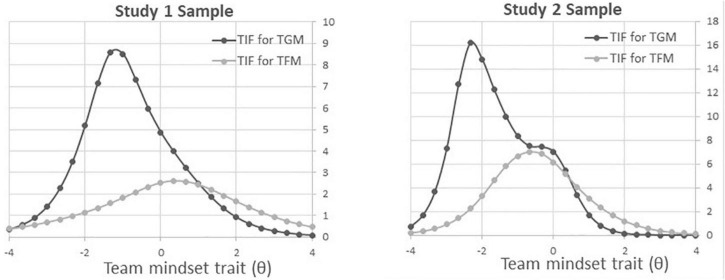
Item response theory (IRT) test information curve for team growth and fixed mindset.

Table 4 lists the eight items of the TMS short-form and the related test properties. Each team growth mindset and team fixed mindset scale had α of 0.90 and 0.77, respectively, and their test information [*T* (θ)] were 24.47 and 27.71 at the middle level of the team mindset (team mindset = 0), as we extended the item information function to the test-level. The team fixed mindset had a bit higher test information than the team growth mindset and as the test information curves in Figure 2. Such test information patterns of team mindsets were similar for Study 1 and Study 2.

**TABLE 4:** Team mindset items for short team mindset scale (TMS).

Items	Team mindset (α = 0.81)
**Team growth mindset [α = 0.90,T(θ) = 24.47]**	
Item 1 (TGM_Change1)	Our team always looked for better ideas
Item 2 (TGM_Risk2)	Our team was enthusiastic and said, “let’s go for it”
Item 3 (TGM_Desire1)	Our team benefited from learning each other’s opinions
Item 4 (TGM_Desire4)	Our team can achieve more collectively than an individual can
**Team fixed mindset [α = 0.77,T(θ) = 27.71]**	
Item 5 (TFM_Change2)	Our team felt that contrary opinions made us less efficient
Item 6 (TFM_Risk1)	Our team relied on a key individual to solve challenges
Item 7 (TFM_Feedback3)	Our team kept one solution throughout the whole time
Item 8 (TFM_Desire4)	Our team can achieve more by letting the leader lead than we can by figuring it out collectively

#### 3.2.4. Factorial invariance on short-form of TMS

The current study had the datasets representing the different numbers of male and female participants, and the participants for Study 1 and Study 2 were from educational settings and workplaces, respectively. However, the measurement needed to be consistent across different gender groups or settings. In other words, regardless of gender and the group from the different settings, individuals with an identical level of latent team mindsets should have an identical conditional probability for the observed team mindset score given the latent level. To verify the equivalent measurement across gender and samples for Study 1 and Study 2, we set the 2-factor model representing team growth mindset and team fixed mindset and tested factorial invariance across sub-groups (Vandenberg and Lance, 2000; Millsap, 2012). The ML estimator was used throughout *Mplus* 8.4 (Muthén and Muthén, 1998–2017) for the analyses.

For the gender group, two datasets of Study 1 and Study 2 were combined, and the factorial invariance between males (*N* = 390) and females (*N* = 575) was examined. From a configural invariance model to a scalar invariance model, more parameters were identically constrained between groups. Table 5 presents the model fit of testing factorial invariance. Based on the alternative fit indices (i.e., CFI, RMSEA, and SRMR), we concluded that the general model fit was enough to hold scalar invariance (CFI = 0.95, RMSEA = 0.06, SRMR = 0.05). In other words, there was no measurement bias on the short-form of TMS regarding different gender groups. In addition, the same analysis was conducted across two samples from Study 1 (*N* = 908) and Study 2 (*N* = 91). As more parameters were equally constrained, the global fit was slightly worsened; however, all alternative fit indices were acceptable at the scalar invariance level (CFI = 0.94, RMSEA = 0.06, SRMR = 0.05). Therefore, factorial invariance hold across samples from different settings as well as gender.

**TABLE 5:** Model fits of factorial invariance test.

Model	χ^2^*(df)*	CFI	RMSEA	SRMR
**Gender (male/female)**				
Configural	96.67**(38)	0.97	0.06	0.04
Metric	118.13**(44)	0.96	0.06	0.05
Scalar	142.96**(50)	0.95	0.06	0.05
**Sample (Study 1/Study 2)**				
Configural	116.63**(38)	0.96	0.06	0.04
Metric	128.60**(44)	0.96	0.06	0.05
Scalar	169.28**(50)	0.94	0.06	0.05

**p < 0.01.

## 4. Discussion

Due to the practical issue regarding the test length of 48-item TMS (Han and Kim, 2020), the current study aimed to develop a short-form of TMS that could as performed and had adequate psychometric properties as the long-form. We investigated the items from various perspectives to select the desired length of TMS. Study 1 tested the dimensionality of the TMS with EFA based on the target rotation. Items were preferred for the short-form of TMS when they showed strong relationships with the target team mindset and justified the hypothesized relationship. For example, the factor loadings were expected to be positive and negative on the team growth mindset and the team fixed mindset respectively. Study 2 examined item-level properties. Items were considered appropriate when they had less floor and ceiling effects but had representative item discrimination and information on the team mindsets. For the final set of TMS items, items repeatedly showed suitable psychometric properties in both studies were selected; the 8-item TMS, four items for each team mindset, was set for the short-form of TMS.

The TMS was examined with the students’ data in Study 1 and the workplace data in Study 2. The student data had a large sample size, so parameter estimates were relatively stable (Embretson and Reise, 2000). However, the information about the team characteristics, such as team membership, types of team projects, or tasks were limited. Hence, we conducted Study 2 using a workplace dataset with the team information. Only a small number of participants completed the survey because of the COVID-19 pandemic. Therefore, we used the robust estimator in Study 2 and separately conducted the item-level analyses given the unidimensionality assumption of team mindsets to lessen the possible estimation issues due to the small sample size. To complement each of the studies, we selected the final set of items when they showed appropriate performances across the two studies.

We found the ceiling effect of the team growth mindset as many participants responded to the strongly agree category. It indicated that people quickly answered the corresponding items regarding the team growth mindset (Allen and Yen, 2001). Although they had a low level of perception of the team growth mindset, they could easily identify the team’s growth mindset with the TMS (Zanon et al., 2016). We tried to lessen the ceiling effect by excluding items with high response rates in the last category. However, the short-form TMS still had the highest test information on the left side of the test information function distribution, as presented in Figure 2. On the other hand, the team fixed mindset items had the highest value in the middle of the test information function distribution. Follow-up studies need to replicate validations on the short-form of TMS with different samples and compare the results to the current study.

Although the present study was at the early stage of measuring the perception of the team’s mindsets, it makes meaningful contributions to the team study. First, it extends mindset research to the context of teams and their mindsets. Second, it offers a practical team mindset instrument for teams in education. The short-form of TMS has a shorter length but has appropriate psychometric properties. Therefore, the current TMS would be useful to measure the team growth and fixed mindsets without the issue derived from a lengthy instrument.

Some limitations need to be further studied. First, the test information curve for the team growth mindset had bimodal information distribution. The possible reason is the combination of the small sample size and participants’ tendency toward ‘*agree*’ categories [sum of category (4) somewhat agree and category (5) strongly agree]. We expect a cross-validation study with a larger sample would verify this aspect later. Second, the test information of the team fixed mindset was smaller than the team growth mindset’s, that is the test information curve of team fixed mindset occupied a smaller area as shown in Figure 2. It is known that the item slope is positively related to item information, and a sum of the item information indicates the test information (Baker, 1985; Embretson and Reise, 2000). Therefore, the lower item slopes might be related to not only the smaller item information, but also the smaller test information. The item slope represents the item discrimination power; hence, revising the item sets might be needed to improve the discrimination power of the items across team fixed mindset levels. Lastly, the current study has not examined relationships between the short-form of TMS and other external variables. We encourage future studies to explore various relationships with other constructs and demographic characteristics to examine how perception of team’s mindsets is related to the other constructs within individuals or teams.

## Data availability statement

The raw data supporting the conclusions of this article will be made available by the authors, without undue reservation.

## Ethics statement

The studies involving human participants were reviewed and approved by the Boise State University. The patients/participants provided their written informed consent to participate in this study.

## Author contributions

All authors listed have made a substantial, direct, and intellectual contribution to the work, and approved it for publication.
